# Ginseng: An Nonnegligible Natural Remedy for Healthy Aging

**DOI:** 10.14336/AD.2017.0707

**Published:** 2017-12-01

**Authors:** Yong Yang, Changhong Ren, Yuan Zhang, XiaoDan Wu

**Affiliations:** ^1^Department of Herbal Formula Science, Chinese Medicine College, Beijing University of Chinese Medicine, Beijing, 100029, China; ^2^Institute of Hypoxia Medicine, Xuanwu hospital, Capital Medical University, Beijing, 100053, China

**Keywords:** Ginseng, Anti-aging, pharmacology, molecular mechanism

## Abstract

Aging is an irreversible physiological process that affects all humans. Numerous theories have been proposed to regarding the process from a Western medicine perspective; however, ancient Chinese medicine practices and theories have increasingly gained attention, particularly ginseng, a grass that has been studied for the anti-aging properties of its active constituents. This review seeks to analyze current data on ginseng and its anti-aging properties. The plant species, characteristics, and active ingredients will be introduced. The main part of this review is focused on ginseng and its active components with regards to their effects on prolonging lifespan, the regulation of multiple organ systems including cardiovascular, nervous, immune, and skin, as well as the anti-oxidant and anti-inflammatory properties. The molecular mechanisms of these properties elucidated via various studies are summarized as further evidence of the anti-aging effects of ginseng.

Aging is an inevitable biological process that leads to progressive structure modification and physiological dysfunction. According to Zhores Medvedev, there are more than 300 hypotheses of aging [1]. Many theories attempt to explain the process of aging, but none seem to be comprehensive [2]. The theory of aging can essentially be categorized into two main schools of thought as to the underlying factors: programmed factors vs. damage-related factors. Programmed factors follow a biological timetable, perhaps a continuation of the one that regulates childhood growth and development. Changes in gene expression subsequently affect the systems responsible for the responses involved in maintenance, repair and defense. Damage-related factors, on the other hand, include internal and environmental assaults to the living organism that induce cumulative damage at various levels [3]. Both theories conclude that the aging body is unable to maintain its normal function and constitution, ultimately leading death. While humans are now living longer, we are not necessarily living healthier. Recent evidence has shown the promise of herbal medicine in protecting against aging and aging related pathologies. LW-AFC, prepared from Liuwei Dihuang decoction, a well-known Chinese herbal medicine believed to delay senescence, has been proven to be beneficial against the deterioration of memory and learning, and play a role in regulating N-glycan in Senescence-Accelerated Mouse Prone 8 Strain, a Mouse Model of Alzheimer’s Disease [4]. Another Chinese herbal medicine, Danggui-Shaoyao-San (DSS), has been shown to have anti-inflammatory and anti-oxidant properties, and reduce cell apoptosis in the hippocampus of free radical-mediated neurological diseases. DSS also participate in the regulation of central nervous systems by improving monoaminergic, cholinergic, dopaminergic, adrenergic, and serotonergic neurotransmission. Moreover, it can modulate cognitive dysfunction in patients with Alzheimer's disease (AD) [5]. Recent studies indicate that DSS attenuate ischemia-induced brain injury in middle cerebral artery occlusion rats and facilitate focal angiogenesis and neurogenesis [6]. Ginseng is a popular herb that has been used in Chinese Medicine for over 2000 years, with its first use records in the ancient Chinese Meteria Medica *ShenNongBenCaoJing (Patron of Agriculture’s Herbal Classic)*. It is believed to maintain vigor and vitality, particularly with regards to strength and intelligence. Many studies have sought to discover the pharmacological effects and mechanisms of this mysterious plant. It has been proven that ginseng indeed possess properties that combat aging, diabetes, cancer, as well as immunoregulatory effects that help with wound and ulcer healing. This review will discuss pharmacological activity of ginseng associated with its anti-aging properties.

## 1. Introduction of ginseng

### 2.1 Species of ginseng

The English word “ginseng” stem from the Chinese word rénshēn. Rén means person, while shēn means plant root. Ginseng’s pronunciation comes from Cantonese “yun sum” or the Hokkien pronunciation "jîn-sim". Ginseng is a slow-growing perennial plant with fleshy roots, and belongs to the genus Panax of the family Araliaceae. The genus Panax derives its name from the Greek words pan (all) and akos (healing). Ginseng is commonly used as a health supplement, and in many herbal formularies to treat all kinds of illnesses in East Asian countries [7]. There are a total of 13 species that grow widely in Asia, North America and Europe [8]. Commonly, ginseng is referred to the dry root and rhizome of Panax ginseng C.A.Meyer (Araliaceae), which is originally grow typically in cooler climates like Northeast China, Korea peninsula and Russia and North America [9, 10]. Panax ginseng include three different species of Korean, Chinese, and American ginseng, which have been globally cultivated and traded [10].

### 2.2 Plant characteristics

Ginseng is a perennial umbel plant, often with a branched,rootstock and a stalk bearing 2-5 whorls and long-stalked leaves with jagged edges. It is a self-pollination plant that bloom at its third-year growth phase. Once the flowers bloom in May, then they develop into red berries, with each fruit containing two seeds. At the time of collecting, seeds are immature without a clear embryo shape, and need a quiescent stage to mature to germination. During cultivation in the latter half of July, the harvest seeds are set in moist, shaded soil for approximately 100 days, and sown during the first half of November and transplanted during the next spring. Ginseng roots are usually harvested at 4 to 6 years of age during autumn. The roots consist of three parts, namely the rhizomes (neck), primary roots and rootlets. A matured 6-year-old ginseng on average would have a total root length of about 34 cm, with the primary root being about 7-10 cm long and 3 cm wide, with several stout rootlets. Average weight ranges from 70-100 g. New buds grow from its rhizome each spring, and the stalk withers away each autumn but traces of it remain in the rhizome each year. The annual stalk growth from ginseng’s rhizome is an important identifier in distinguishing ginseng from other ginseng-related products [11, 12].

### 2.3 Processing of ginseng

Ginseng’s medical products are classified into three categories - fresh, white, and red ginseng depending on the processing [9]. Almost all medical products are derived from ginseng after 4 to 6 years of cultivation. Fresh ginseng is defined as less than 4-year-old and required minimal processing. White ginseng is 4-6-year-old, and is peeled and dried. Red ginseng is 6 years old that is first steamed and then dried. Each type of ginseng can be further processed into different formulations, including powder, extract (tinctured or boiled), juice, tea, capsules, tablets, and more [8]. Red ginseng is considered to be more effective since heating inactivate its catabolic enzymes, thereby restraining its overall deterioration while increasing antioxidant-like substances that suppress lipid peroxide formation, and improve gastrointestinal absorption. Heating red ginseng has been proven to degrade the thermally unstable malonyl-ginsenoside into proportionable neutral ginsenosides, causing degradation or transformation of neutral ginsenosides and reducing acidic malonyl-ginsenosides. The hydrolyzed ginsenosides in red ginseng is responsible for its multiple regulatory effects including inhibition of cancer cell growth via apoptosis, antimetastatic activities, vasorelaxation properties, and anti-platelet aggregation activity [13]. Data from experiments on the standardization and optimization of red ginseng preparation allow for guaranteed quality [14]. Newer methods that improve product efficacy include fermentation [15, 16] or organic acid pretreatment [17, 18]. A new study has shown that pectinase-treated Panax ginseng improves hydrogen peroxide-induced oxidative stress in GC-2 sperm cells and regulates testicular gene expression in aged rats [19].

### 2.4 Active part and ingredients

Traditionally, the root of ginseng is considered to be the only effective part used to treat various conditions, but through phytochemical studies and pharmacological research, its other parts including the flowers, leaves and fruits have also been discovered to be effective against fatigue, hyperglycemia, obesity, cancer, and possess anti-oxidant, anti-inflammation and anti-aging properties[20, 21]. Approximately 200 substances have been isolated from Korean ginseng [22], and 100 substances from American ginseng thus far[21]. Recent phytochemistry and pharmacological studies have discovered a variety of potent components in all parts of the ginseng plant including ginsenosides, alkaloids, phenolics, phytosterol, carbohydrates, polypeptides, ginseng oils, amino acids, nitrogenous substances, vitamins, minerals, and certain enzymes. Ginsenosides are the major bioactive metabolites [23, 24]. There are a total of 38 ginsenosides in Panax ginseng C A Meyer (Korean ginseng) and 19 ginsenosides in Panax quinquefolius L (American ginseng) [11]. Ginsenosides are triterpene saponins. Most consist of a dammarane skeleton (17 carbons in a four-ring structure) with different sugar groups (e.g. glucose, rhamnose, xylose and arabinose) connected to the C-3 and C-20 positions [25, 26]. Ginsenosides are nominated as 'Rx', where the 'R' represents the root and the 'x' describes the chromatographic polarity in an alphabetical order [27]. For instance, Ra is the least polar compound, and Rb is more polar than Ra. Ginsenosides can be classified as dammarane-type, ocotillol-type and oleanane-type oligoglycosides [28]. Dammarane-type saponins can be further classified into protopanaxadiol (PPD like Rb1, Rb2, Rb3, Rc, Rd, Rg3, Rh2, Rs1) and protopanaxatriol (PPT like Re, Rf, Rg1, Rg2, Rh1) types [21, 26, 29, 30]. The chiral carbon C-20 position in PPD and PPT type can be substituted for isobutyl, and is further divided into 20 (S) and 20 (R). So far, more than 70 ginsenosides have been separated from the three main kinds of ginseng. Among them, ginsenosides Rbl, Rb2, Rc, Rd, Rgl, Rg2, and Re are the major constituents of white and red ginseng, while ginsenosides Rg3, Rg5, and Rg6 are unique to red ginseng [31]. Some rare ginsenosides, like the ocotillol saponin F11 (24-R-pseudoginsenoside) [32] and the pentacyclic oleanane saponin Ro (3,28-O-bisdesmoside) [33] have also been isolated and identified. The quality and composition of ginsenosides in the ginseng plants are influenced by a number of factors including the species, age, part of the plant, cultivation method, harvesting season and storage method [34, 35]. Using ginsenoside Rf as example, Rf is exclusive to Asian ginseng whereas F11 is unique to American ginseng. Thus the Rf/F11 ratio is applied as a phytochemical label to differentiate American from Asian ginseng [36, 37]. Many reports indicate that ginsenoside metabolites show better biological effects than ginsenosides. For example, Rh2 and PD, metabolites of Rg3, have more potent anti-tumor activities than ginsenoside Rg3. Unlike Ginsenosides Rb1, Rb2, Rg1 and Re, compound K, PT and PD, the intestinal metabolites of PPTs and PPDs, have inhibitory effects similar to that of the human liver enzyme cytochrome P450 inhibitory effects [38, 39].

## 3 Anti-aging properties of ginseng

As aging is a multisystem and multifactorial process, the different theories of aging are actually contradictory. In this review, we present evidence of the anti-aging properties of ginseng discovered through research, and how the evidence largely supports the damage or error theory as aforementioned.

### 3.1 Prolonging lifespan

Historically, ginseng has been thought to prolong lifespan. Recently, several studies have shown that the components of ginseng can prolong the life span of experimental models such as *Drosophila* and *C.elegans* [40, 41]. While ginseng does not significantly prolong the lifespan of aging mice, but it stabilize mice’s behavior by antagonizing stress [42]. In one study, CVT-E002, a proprietary extract from North American ginseng, extends the life span of infant and juvenile mice with leukemia in a dose-dependent manner, with a limit of 20 mg/day [43].

### 3.2 Anti-oxidation effects of ginseng

Increase in oxygen-derived free radicals is closely related to the aging process. Reactive oxygen species are produced by intracellular molecular pathways located mainly in the cytoplasm and mitochondria [44]. Korean red ginseng decreases lipid peroxidation and restores anti-oxidant potential by reducing oxidative stress in rats. Aged rats fed with a Korean red ginseng water extract diet exhibited much less oxidative damage [45, 46].Ginseng’s anti-oxidant effects have also been clinically proven. In a double-blind randomized controlled clinical trial, administration of Korean ginseng led to a significant decrease in the levels of serum reactive oxygen species (ROS) and methane dicarboxylic aldehyde (MDA), while potentiating the total glutathione content and glutathione reductase (GSH-Rd) activity [47]. Other studies have focused on oxidation induced organ injury. Fermented Panax ginseng extract administration to aged rats resulted in enhanced activities of superoxide dismutase e(SOD), glutathione peroxidase (GPx), glutathione reductase (GR), catalase (CAT) and glutathione-S-transferase (GST) while increasing glutathione (GSH), ascorbic acid and α-tocopherol levels in the liver, kidneys, heart and lungs [46]. In an experimental mouse model of chronic cyclosporine (CsA) nephropathy, Korean ginseng reduced serum creatinine and blood urea nitrogen and increased creatinine clearance. Proinflammatory and profibrotic molecules such as cytokines, induced nitric oxide synthase, transforming growth factor (TGF)-β1 and TGF-β1-inducible gene h3 and apoptotic cell death were reduced [48]. Another study showed that in an ethanol-induced liver damage rat model, pretreatment with Korean ginseng extract maintained the activity of serum Glutamic Pyruvic Transaminase (GPT), and decrease MDA concentrations [49]. Free radicals play a major role in mediating skeletal muscle damage and inflammation after strenuous exercise and in muscle diseases [50]. Ginseng extract is believed to protect muscle from oxidative stress caused by acute exercise by decreasing MDA level in rats [51]. What components of ginseng play a role in these anti-oxidation effects? Studies of the components in ginseng has been productive and new compounds is continuously being isolated. The chemical constituents isolated from ginseng including polysaccharides, ginsenosides, peptides, polyacetylenic alcohols, fatty acids, etc [9]. IH901 or compound K, is considered to be the final intestinal bacterial metabolite of ginseng in humans. IH901 administration has beneficial effects on moderating exercise-induced oxidative stress and restores antioxidant defense capabilities in rat skeletal muscles and lung tissue [52]. Polysaccharides, another major bioactive constituents of P. ginseng, also is a strong antioxidant [53]. A recent study showed phenolic compounds in white ginseng can effectively trigger antioxidant enzyme activity [54]. The essential oils from ginseng leaves possess weak DPPH (1,1-Diphenyl-2-picrylhydrazyl radical 2,2-Diphenyl-1-(2,4,6-trinitro-phenyl)hydrazyl) and 2,2′-azinobis-(3-ethyl-benzothiazoline-6-sulfonic acid(ABTS) radical scavenging activities [55]. Ginseng’s antioxidant effects hold exciting clinical potential. Red ginseng has the ability to protect cells from AA + iron-induced ROS production and mitochondrial impairment through AMP-activated protein kinase (AMPK) activation. At the molecular level, ginseng triggers LKB1-dependent AMPK, which in turn leads to increased cell survival [56]. Using old rat fatigue model of major small intestinal resection (MSIR), ginsenoside Rb1 counteracts fatigue, the mechanism of which likely involves activation of the PI3K/Akt pathway with subsequent Nrf2 nuclear translocation and induction of antioxidant enzymes [57].

### 3.3 Cardiovascular effects of ginseng

Aging is associated with various, complicated and changes in cardiovascular structure and function. The heart becomes slightly hypertrophic and has a dampened response to sympathetic stimuli, including increase in heart rate and myocardial contractility. The aorta and central elastic arteries become dilated and stiff, exhibiting enhanced pulse wave velocity, endothelial dysfunction and biochemical transformation that resembles early atherosclerosis [58]. To compensate for the decrease in arterial compliance and increase in peripheral resistance, the heart must pump with greater force. The myocardium responds in much the same way as other muscles do after exposure to increased load - enlargement and hypertrophy that result in a gradual increase in cardiac weight [59]. There is a gradual decrease in cardiac myocytes, while remaining myocytes become hypertrophic and the myocardium shows increased levels of collagen [58]. Ginseng has been shown to be cardioprotective through its anti-oxidative, anti-arrhythmic, calcium channel-antagonistic, anti-inflammatory and anti-apoptotic properties [60]. Acute administration of ginseng resulted in depressed cardiac contractile function, as evidenced by reductions in HR and blood pressure that persisted up to 24 h. This acute reduction in cardiac contractility appears to be intrinsic to the myocardium, as spontaneously beating perfused hearts exposed to different concentrations of North American ginseng also demonstrated inhibitory response [61]. On the contrary, Panax ginseng dramatically increases cardiac contractility in normal rats [62]. The different in response in contractility may due to the differences in components in ginseng of different origin. For instance, in North American ginseng, ginsenosides Rb1 and Re appear to be most abundant, whereas Asian ginseng is enriched more in Rg1 and Rg2 ginsenosides [61]. In intermediate-aged rats, long term consumption of ginseng extract decreased the sensibility of hearts to acute ischemia reperfusion injury. These effects might be regulated through the activation of Akt/eNOS, inhibition of Erk/caspase7, and upregulation of Sirt1 and Sirt3. A study on American ginseng also showed its protection of myocardium from ischemia and reperfusion injury via the upregulation of endothelial nitric oxide synthase [63]. In addition to the effects on myocardium, a clinical investigation revealed that Korean red ginseng may improve arterial stiffness determined by augmentation index (AI). It appears that ginsenosides, rather than polysaccharides, may be the major pharmacologically active constituent of the root of Korean red ginseng [64].

Another major cardiovascular effect of ginseng is endothelial regulation, which plays an important in role in the alteration of blood vessels with age. The endothelium is the innermost layer of blood vessels that comes into direct contact with the blood. It is composed of a single layer of squamous epithelial cells, which are regular and smooth in children and young adults, offering minimal resistance to blood flow. With age, the endothelial layer starts to have atypically shaped cells and becomes thickened due to smooth muscle fibers that migrate from the tunica media. This thickening not only contributes to a reduction in arterial elasticity and compliance, but also the lumen size, further increasing resistance to blood flow [65]. A clinical trial demonstrates that Korean red ginseng (KRG) and its ginsenosides significantly improved flow mediated vasodilatation post treatment [66]. A recent study showed that KRG inhibited arginase activity, maintained nitric oxide (NO) generation, reduced ROS production, and increased eNOS coupling in aged mice. KRG also improved blood vessel tension, as indicated by increased acetylcholine-induced vasorelaxation and decreased phenylephrine-stimulated vasoconstriction. In addition, KRG reduced plasma peroxynitrite production in aged mice, indicating decreased lipid peroxidation. These results suggest KRG exerts its vasoprotective effects by suppressing arginase activity and enhancing NO signaling [67]. However, cardiovascular remodeling due to age is by no means a uniform and generalized structural degeneration; rather, different components of the cardiovascular system may be affected heterogeneously [58]. More multi-level analyses are needed to evaluate the long-term benefits of ginseng.

### 3.4 Anti-aging effect of ginseng on nervous system and motor function.

There is a progressive loss of neural tissue with age, usually reflected by a gradual decline in cognitive function. With age, cerebral blood flow decreases by around 20% [68].There is an age-related decline in the synthesis of many neurotransmitters and their receptors. These include the catecholamines (adrenaline and noradrenalin), dopamine and serotonin. These reductions can slow reaction time, impair information processing and, sometimes, increase the risk of depression [65]. Neurodegenerative disorders occur when cells fail to react to age-related increases in oxidative, metabolic and ionic stress, consequently resulting in the accumulation of damaged proteins, deoxyribonucleic acid (DNA) and cell membranes [69]. Ginseng has been commonly applied and studied for its enhancement of cognition enhancing and stress reduction. The underlying molecular mechanisms of ginseng’s effects on the brain have been widely studied, and found to involve monoamine transmission, glutamatergic transmission, estrogen signaling, nitric oxide production, the Keap1/Nrf2 adaptive cellular stress pathway, neuronal survival, apoptosis, neural stem cells and neuroregeneration, microglia, astrocytes, oligodendrocytes and cerebral microvessels [70].

Cognition is a fundamental action of the brain that becomes disrupted with aging, and the capabilities of maintaining and processing information decline with age [71]. Memory impairment is considered one of the most predominant downfalls of aging, and thereby has been a major point of research focus [72, 73]. A recent study demonstrated that prolonged Red ginseng administration inhibited the production of age-processed inducible nitric oxide synthase, cyclooxygenase-2, interleukin-1b and tumor necrosis factor-a expressions. Furthermore, the antioxidative properties of red ginseng in aged mice restored glutathione level, and increased antioxidative-related enzymes nuclear factor erythroid 2-related factor 2 (Nrf2) and heme oxygenase-1 (HO-1). These results indicate that age-related decline of learning and memory can be halted through anti-inflammatory mechanisms [74]. A double-blind clinical trial from Korea revealed that Korean ginseng can improve certain psychomotor functions such as as mental arithmetic in healthy subjects, as assessed by different tests of psychomotor performance [75]. Early animal experiments demonstrated that red ginseng improves learning and memory in aged animals. Red ginseng had a more prominent effect on memory than on learning in rats of all ages. In 22-month old rats, red ginseng not only improved memory, but also promote the rapid acquisition of responses [76]. The underlying mechanism of memory improvement has been explored by several studies. Ginseng extracts and ginsenoside Rg1, Rb1 improved acquisition and retention of memory in aged rats, ovariectomized rats, and cerebral ischemia-reperfusion rats [77]. Ginsenoside Rg1 and Rb1 were found to increase cholinergic activity through two signaling pathways. First, both types of ginsenosides increase the density of central M-cholinergic receptors without specifically binding to the receptors. Second, Rg1 and Rb1 increased the level of acetylcholine (Ach) in the central nervous system (CNS), which might be the result of ginsenoside-induced choline acetyl transferase activity and inhibition of acetylcholine esterase activity [78, 79]. As the same time, both ginsenosides also promote cholinergic neurotransmission by increasing cholinergic metabolism in the CNS [80]. Further investigation prompt that Ginseng extract in a dose-dependent manner can improve memory defect through the restored homeostasis via increasing neurotransmitter levels and reducing acetylcholinesterase (AChE) activities in rat brain areas in cortex, hippocampus and striatum [81]. Korean scientists found that gintonin, an isolated novel lysophosphatidic acids (LPAs)-ginseng protein complex derived from ginseng, activating LPA receptor via N-methyl-D-aspartic acid (NMDA) receptor channel activity in Xenopus oocytes, and elevated long term potentiation (LTP) in cultured hippocampal neurons in concentration-dependent manners, which might be responsible for ginseng-mediated improvement of memory-related brain functions [82]. Neural progenitor cells (NPCs) proliferation and differentiation is essential for brain development. They are thereby believed to be a potential focus in the treatment of age-related neurodegenerative diseases. Ginsenoside Rg1 significantly increases the number of proliferating progenitor cells in animal hippocampus, enhances the activity of the antioxidant enzymes glutathione peroxidase (GSH-Px) and superoxide dismutase (SOD), decreases the levels of proinflammatory cytokines such as IL-1b, IL-6 and TNF-a. These positive results suggest that the regulation of proinflammatory cytokines levels, and enhancement of antioxidant activities could be the anti-aging mechanism of ginsenoside Rg1 [83, 84]. In the brain aging animal model, administration of Rg1 also protected neural stem cells (NSCs) as demonstrated by an increased level of SOX-2 expression, reduced astrocytes activation shown by reduced level of Aeg-1 expression, increase in telomere lengths and telomerase activity, and downregulation of mRNA expression of cellular senescence associated genes p53, p21Cip1/Waf1 and p19Arf in the hippocampus. These results imply that ginsenoside Rg1 plays a beneficial role in the regulation of proliferation of hippocampal progenitor cells proliferation, which may be the crux of its anti-aging properties [83]. Further investigation indicate that Rg1 significantly increases the expression of synaptic plasticity-associated proteins in hippocampus in normal old aged mice, including synaptophysin, N-methyl-d-aspartate receptor subunit 1, postsynaptic density-95, and calcium/calmodulin-dependent protein kinase II alpha, by targeting rapamycin pathway activation. This data has provided powerful evidence for Rg1 regulation of cognitive decline during aging [85].

Ginseng and its components, especially ginsenosides, is not only effective in anti-aging, but also beneficial in aging-related neurological disorders, including Alzheimer's disease (AD). In senescence-accelerated mouse prone 8 (SAMP8) mice model, 3 months administration with ginsenoside Rg1 significantly reduced the contents of soluble Aβ_1-40_ in the hippocampus, and decreased hippocampal PKA RIIα (isoform IIα of the regulatory subunit of PKA) levels. Consequently, learning and memory improvement was evident, suggesting long-term application of ginsenoside Rg1 may postpone cognitive decline via increasing Aβ generation, PKA/CREB activity, as well as brain derived neurotrophic factor (BDNF) content in the brain [86]. In addition to ginsenoside Rg1, ginsenoside Rb and Rd also have potent neurological protective and regulatory effects. For instance, Rb fraction protected both astrocytes and neurons, especially GABAergic inter neurons, and preserved microglial homeostasis against kainate-induced excitotoxicity. Rb1 shows its anti-oxidative effects as it can increase embryonic cortex-derived neural progenitor cells (NPCs) and suppress the cell apoptosis int-BHP-induced oxidative injury via Nrf2 pathway activation [87]. In old rats following acute lead (Pb) exposure, ginsenoside Rd reduces of microglial activation and maintains of neural stem cells proliferation [61, 88-90]. PPD, the main intestinal metabolite of ginsenosides, is one of the active ingredients in ginseng, exert neuroprotective effect by triggering antioxidant activation and promoting mitochondrial function that in turn suppress apoptosis to maintain regular neurological activity [91].

Ginseng is also considered to have stress-relieving properties. Its active component, ginsenoside, is similar in structure to estrogen. In immobilization (IMO) stressed mice, red ginseng administration prior to IMO stress down regulated peptidyl arginine deiminase type 4 (PADI4) through up regulation of the estrogen receptor (ER)β expression in the brain, which was in itself up-regulated by various stresses factors such as H2O2, acrylamide, and tunicamycin [92]. Ginseng also significantly enhanced the memory of AD rats, and prolonged platform crossing times and the percentage of residence time in the original platform quadrant of spatial probe test. Ginseng also decreases the content of Aβ1-42 and p-tau and promotes the expression of PI3K, p-Akt/Akt, and Bcl-2/Bax mRNA and protein in the hippocampus [93]. American Ginseng, very similar to Asian Ginseng, has similar neuroprotective properties. Daily consumption of American ginseng improved neurocognitive function in senescence-accelerated mice, which could be related to the upregulation of insulin and ChAT gene expression in the brain [94]. Long-term stress causes massive loss of neurons and cognitive deficits. Mounting evidence argue that accumulation of nitric oxide (NO), a intercellular messenger, plays a vital role in the pathogenesis of memory impairment. Chronic unpredictable stress (CUS) model showed induction of remarkable impairment in both acquisition and retention memory associated with alterations in oxidative stress markers, mitochondrial enzyme complex activities, proinflammatory cytokine (TNF-α), and acetylcholinesterase levels in the hippocampus. Furthermore, a significant increase in serum corticosterone levels was seen. American Ginseng administration decreased TNF-α, acetylcholinesterase and corticosterone levels, and reduced oxidative-nitrergic tension. These results demonstrate that regulation of nitrergic signaling cascade associate with the protective effects of American Ginseng by antagonizing CUS-induced oxidative stress, cognitive dysfunction, and neuroinflammation [95]. In an Aβ1-42-induced AD mice model, cognitive function was recovered by oral administration of Cereboost™, the main ingredients being ginsenoside Rb1 derived from American Ginseng. Moreover, Cereboost™ recovered brain microtubule-associated protein 2 and synaptophysin as well as acetylcholine concentration. The data suggest that ginsenoside Rb1 restored the cognitive function of AD model mice by increasing acetylcholine levels via ChAT gene expression [96]. These results suggest that Na+ channel block by American ginseng extract and Rb1 was primarily due to interaction with the inactive state of the channel. Inhibition of the Na+ channel activity by American ginseng extract may contribute to its neuroprotective effect during ischemia [97].

Motor function disorder is another important manifestation of aging. Ginseng has been proved experimentally to also affect motor function [98, 99]. Research on the spontaneous motor activity and central dopaminergic systems in old rats with ginseng interference showed that oral intake of a water extract of Panax ginseng for a certain period of time induced an increase in spontaneous motor activity during the dark and a remarkable reduction of spontaneous motor activity during the daytime. These results indicate that ginseng extract intake may affect the activity of nigro-striatal dopamine neurons via inhibition during the daytime and activation during the dark in old rats [99]. Ginseng and its extracts have been demonstrated to protect experimentally demonstrate skeleton muscle protection from damage due to excess exercise, likely by reducing the release of myocellular proteins, lipid peroxidation and inflammation [51, 100, 101].

### 3.5 Skin effects of ginseng

The aging process is often visible in the skin. Several factors are involved in the aging of skin, including genetics, environmental stress, hormonal alterations and metabolism, all of which lead to accumulative transformations in skin constitution, function and appearance [102]. With age, skin becomes thin, dry, and loses its elasticity and structural regularity. A reduction in the number of fibroblasts in the extracellular matrix result in general atrophy. Decreased levels of collagen and elastin are mainly the results of suppressed protein synthesis affecting types I and III collagen in the dermis, associated with a loss of extracellular matrix proteins and wrinkle formation Oxidative stress is also considered to be a primary driver in the aging process [102]. Panax ginseng and ginsenosides have shown to prevent aging of skin. Two separate clinical trials indicated that ginseng extract improved facial wrinkling [103, 104]. Fermented red ginseng is believed to be more effective in reducing wrinkles and enhance whitening compared to unfermented red ginseng [105]. The reduction in wrinkles may be due to an increase in type I procollagen synthesis [104]. Ginseng and can protect against UV radiation-induced skin damage The skin of the UV-irradiated mice manifested characteristic signs of photoaging, such as deep wrinkles across the back, increased epidermal thickness, cell infiltration, and many enlarged keratinizing cysts, which remarkably improved with red ginseng administration [106]. ginsenoside Rb1 (100 fg, 10 pg, or 1 ng/mouse, topical application) reduced the increase in skin wrinkling, thickness, and epidermis in UVB-irradiated skin aging in hairless mice [107]. The mechanism of wrinkle inhibition in chronic UVB irradiation in hairless mice with red ginseng extract may be due to an increase in the protein level of procollagen type I, unlike observations obtained through clinical trials [105]. MMP-1mRNA and protein levels were significantly downregulated. This data indicates that the anti-wrinkle effect of Korean red ginseng involved the suppression of collagen degradation, rather than increased collagen synthesis [108]. Further investigation showed that one of the stereoisomeric forms of Ginsenoside Rh2, 20(S)-ginsenoside Rh2 [20(S)-Rh2], was able to suppress UV-B-induced ROS production with inhibition of MMP-2 activity and expression in HaCat cells [109]. Hyaluronan is considered to play a major role in the organization of the extracellular matrix structure, as well as the defense responses in human skin cells. Ginseng can also the upregulate hyaluronic acid generation [110, 111]. 20-O-β-D-glucopyranosyl-20 (S)-protopanaxadiol (compound K), one of the major metabolites of ginsenosides, triggered a two-fold increase in hyaluronan synthase2 (HAS2) gene expression in HaCaT cells and induced significant hyaluronan synthesis in human skin cells and hairless mouse skin [110]. 20GPPD is the primary bioactive metabolite of Rb1. In human keratinocytes, 20GPPD increased HA production by elevating hyaluronan synthase 2 (HAS2) expression as an upstream regulator of ERK and Akt activity [111]. The ingredients of ginseng leaves inhibit ROS generation, GHS depletion, and expression of MMP-2 and MMP-9 induced by UVA irradiation in human keratinocyte cells (HaCaT) [112]. The anti-melanogenic activity of ginseng berry extract in skin was strongly associated with the activation of the longevity gene foxo3a [113].

### 3.6 Immuno-regulatory and anti-inflammatory effects of ginseng

As the adaptability of the immune system declines accordingly with age, it is less able to respond properly to invasion of foreign agent. Such changes in defenses result in immune vulnerability [114]. Both Korean and American ginseng have been reported to have immune-regulatory properties [115-118]. In a clinical trial, Y-75 (Ginsan), an acidic polysaccharide extracted from Korean Panax ginseng, was shown to be an immunomodulator that significantly increased NK cell cytotoxic activity, and enhanced phagocytic activity of peripheral blood cells as well as serum TNF-α levels [115]. American ginseng root polysaccharides (AGRPS) stimulates immune function while simultaneously inhibiting the response to lipopolysaccharide (LPS)-induced proinflammatory response [116]. Ginsenoside Rg1 stimulates lymphocyte proliferation drawn from 10 young and 19 elderly patients, and increased the lymphocyte membranes fluidity [117]. In mice forced to undergo a swimming test, P. ginseng was shown to boost immunity via cytokine production of T cells in mouse peritoneal macrophages [118]. Another study with Ginseng polysaccharide,an isolated bioactive constituent from the root of North American ginseng or Panax ginseng C.A. Meyer,was found to induce the proliferation of T cells and B cells, activate macrophages to produce reactive nitrogen intermediates and release some anti-tumor cytokins as TNF-α and IL-6 [119]. Another investigation showed that ginseng can initiate a transcriptional profile of immunoregulation featuring a net T(h)1 immune response, by up-regulating multiple pro-inflammatory cytokines while down-regulating TGF-β, IL-13 and the lipopolysaccharide (LPS) co-receptor CD14, the pivotal signaling pathways might involve the MAPK (ERK-1/2), PI3K, p38 and NF-κB cascades [120]. Ginsenoside Rg1 treatment was also proved significantly up regulate tumor necrosis factor (TNF)-α, but down regulate interleukin-6 (IL-6) protein expression in both lipopolysaccharide (LPS)-activated RAW 264.7 cells and mouse peritoneal macrophages, which support a novel mechanism for Rg1 immune-regulation in macrophages via the NF-κB and PI3K/Akt/mTOR pathways [121]. Korean red ginseng extract (RGE) is considered one of the most common herbs that regulate the immune system. RGE inhibited IL-1β maturation modulated with the nucleotide-binding domain leucine-rich repeat containing (NLR) family, as well as that of pyrin domain-containing 3 (NLRP3) inflammasome activation, and reduced IL-1β secretion and pathogen clearance through pyroptotic cell death by macrophages via inhibition of AIM2 inflammasome activation [122]. Shenzao Cha (SZC), a herbal combination of American ginseng and Chinese jujube, notably enhances spleen and thymus indices and T-lymphocyte proliferation, increase NK cell activity, demonstrating its potent immune-regulatory effects on both innate and adaptive immunity in healthy ICR mice [123]. The extracts from Ginseng Radix et Rhizoma can also improve immune function in rats [124].Dammarane triterpenes compounds from leaves of panax ginseng significantly upregulated interleukin-12 expression in LPS-activated mouse peritoneal macrophage, enhanced the Th1 response-mediated cytokine IL-2, and decreased Th2 response-mediated cytokines IL-4 and IL-6 expression on ConA-activated splenocytes, suggesting a stronger effect on cellular immunity [125]. Red ginseng acidic polysaccharide (RGAP), apharmacological ingredient of Korean red ginseng, shows immune-stimulatory and anti-tumor activity through activating macrophage function by stimulating transcriptional factors including NF-κB and AP-1 and their upstream signaling enzymes, such as ERK and JNK [126]. American ginseng root polysaccharides (AGRPS) stimulate innate immune function while inhibiting lipopolysaccharide (LPS)-induced pro-inflammatory response [116]. Recent research has proposed the concept of chronic inflammation as a major risk factor for aging and age-related diseases, especially low-grade, unresolved, molecular inflammation which may be the deciding factor in development of age-related pathologies [127]. There has been much recent interesting in the anti-inflammatory effects of ginseng. In inflammatory skin diseases such as atopic dermatitis, ginseng extracts inhibit cytokines such as TNF-α and IL-8 in LPS-stimulated human keratinocytes in human dermal fibroblasts [128]. Panax ginseng extract suppresses exercise-induced muscle injury and inflammation by reducing plasma creatine kinase activity (CK) and interlukin-6 (IL-6) levels [101]. American ginseng extract not only prevents but also treats colitis, likely due to a mechanism involving the down-regulation of inducible nitric oxide synthase and cyclooxygenase-2 (markers of inflammation), as well as induction of apoptosis of inflammatory cells via the p53 pathway [129, 130]. Ginseng of different origin have been proven to also inhibit cardiac hypertrophy and atherosclerosis, both of which are closely related aging and aging related disease [131-133].

## 4.Conclusion

Aging is a complicated process with multiple modulations occurring at many levels, from the molecular to the cells. Ginseng, an ancient Chinese herb widely used in Eastern medicine, has been studied for its anti-aging properties., and has been shown to have beneficial effects with regards to anti-inflammation, anti-oxidation, cardiovascular regulation, neurological improvement, anti-tumor, skin protection and immune modulation. The evidence on the life-prolonging effects of ginseng remains inadequate, and further studies are recommended. Investigations integrating science and technology will be needed to further explore the effects of ginseng on the human body to fully understand its potential.

## References

[1] Medvedev ZA (1990). An attempt at a rational classification of theories of ageing. Biol Rev Camb Philos Soc, 65: 375-398 2205304 10.1111/j.1469-185x.1990.tb01428.x

[2] Davidovic M, Sevo G, Svorcan P, Milosevic DP, Despotovic N, Erceg P (2010). Old age as a privilege of the "selfish ones". Aging Dis, 1: 139-146 22396861 PMC3295027

[3] Jin K (2010). Modern Biological Theories of Aging. Aging Dis, 1: 72-74 21132086 PMC2995895

[4] Wang J, Cheng X, Zeng J, Yuan J, Wang Z, Zhou W, et al (2017). LW-AFC Effects on N-glycan Profile in Senescence-Accelerated Mouse Prone 8 Strain, a Mouse Model of Alzheimer's Disease. Aging Dis, 8: 101-114 28203484 10.14336/AD.2016.0522 PMC5287383

[5] Fu X, Wang Q, Wang Z, Kuang H, Jiang P (2016). Danggui-Shaoyao-San: New Hope for Alzheimer's Disease. Aging Dis, 7: 502-513 27493835 10.14336/AD.2015.1220 PMC4963193

[6] Ren C, Wang B, Li N, Jin K, Ji X (2015). Herbal Formula Danggui-Shaoyao-San Promotes Neurogenesis and Angiogenesis in Rat Following Middle Cerebral Artery Occlusion. Aging Dis, 6: 245-253 26236546 10.14336/AD.2014.1126 PMC4509473

[7] Xiang YZ, Shang HC, Gao XM, Zhang BL (2008). A comparison of the ancient use of ginseng in traditional Chinese medicine with modern pharmacological experiments and clinical trials. Phytother Res, 22: 851-858 18567057 10.1002/ptr.2384

[8] Yun TK (2001). Brief introduction of Panax ginseng C.A. Meyer. J Korean Med Sci, 16 Suppl: S3-5 11748372 10.3346/jkms.2001.16.S.S3 PMC3202213

[9] Ru W, Wang D, Xu Y, He X, Sun YE, Qian L, et al (2015). Chemical constituents and bioactivities of Panax ginseng (C. A. Mey.). Drug Discov Ther, 9: 23-32 25788049 10.5582/ddt.2015.01004

[10] Shin BK, Kwon SW, Park JH (2015). Chemical diversity of ginseng saponins from Panax ginseng. J Ginseng Res, 16: 287-298 10.1016/j.jgr.2014.12.005 PMC4593792 26869820

[11] Choi KT (2008). Botanical characteristics, pharmacological effects and medicinal components of Korean Panax ginseng C A Meyer. Acta Pharmacol Sin, 29: 1109-1118 18718180 10.1111/j.1745-7254.2008.00869.x

[12] Bown D, editor. Encyclopedia of Herbs. London: Dorling Kindersley Limited; 2008.

[13] Nam KY (2005). The Comparative Understanding between Red Ginseng and White Ginsengs, Processed Ginsengs (Panax ginseng C. A. Meyer). J Ginseng Res, 29: 1-18

[14] Yang SJ, Woo KS, Yoo JS, Kang TS, Noh YH, Lee JS, et al (2006). Change of Korean Ginseng Components with High Temperature and Pressure Treatment. Korean J Food Sci Technol, 38: 521-525

[15] Kim HJ, Lee SG, Chae IG, Kim MJ, Im NK, Yu MH, et al (2011). Antioxidant effects of fermented red ginseng extracts in streptozotocin- induced diabetic rats. J Ginseng Res, 35: 129-137 23717054 10.5142/jgr.2011.35.2.129 PMC3659529

[16] Kim BG, Choi SY, Kim MR, Suh HJ, Park HJ (2010). Changes of ginsenosides in Korean red ginseng (Panax ginseng) fermented by Lactobacillus plantarum M1. Process Biochem, 45: 1319-1324

[17] Kim MH, Hong HD, Kim YC, Rhee YK, Kim KT, Rho JH (2010). Ginsenoside Changes in Red Ginseng Manufactured by Acid Impregnation Treatment. J Ginseng Res, 34: 93-97

[18] Yi JH, Kim MY, Kim YC, Jeong WS, Bae DW, Hur JM, et al (2010). Change of ginsenoside composition in red ginseng processed with citric acid. Food Sci Biotechnol, 19: 647-653

[19] Kopalli SR, Cha KM, Jeong MS, Lee SH, Sung JH, Seo SK, et al (2016). Pectinase-treated Panax ginsengameliorates hydrogen peroxide-induced oxidative stress in GC-2 sperm cells and modulates testicular gene expression in aged rats. J Ginseng Res, 40: 185-195 27158240 10.1016/j.jgr.2015.08.005 PMC4845052

[20] Jiao L, Li B, Wang M, Liu Z, Zhang X, Liu S (2014). Antioxidant activities of the oligosaccharides from the roots, flowers and leaves of Panax ginseng C.A. Meyer. Carbohydr Polym, 106: 293-298 24721081 10.1016/j.carbpol.2014.02.035

[21] Kim DH (2012). Chemical Diversity of Panax ginseng, Panax quinquifolium, and Panax notoginseng. J Ginseng Res, 36: 1-15 23717099 10.5142/jgr.2012.36.1.1 PMC3659563

[22] Attele AS, Wu JA, Yuan CS (1999). Ginseng pharmacology: multiple constituents and multiple actions. Biochem Pharmacol, 58: 1685-1693 10571242 10.1016/s0006-2952(99)00212-9

[23] Kim YJ, Zhang D, Yang DC (2015). Biosynthesis and biotechnological production of ginsenosides. Biotechnol Adv, 33: 717-735 25747290 10.1016/j.biotechadv.2015.03.001

[24] Hou JP (1977). The chemical constituents of ginseng plants. Comp Med East West, 5: 123-145 608333 10.1142/s0147291777000209

[25] Tanaka O, Han EC, Yamaguchi H, Matsuura H, Murakami T, Taniyama T, et al (2000). Saponins of plants of Panax species collected in Central Nepal, and their chemotaxonomical significance. III. Chem Pharm Bull (Tokyo), 48: 889-892 10866157 10.1248/cpb.48.889

[26] De Smet PA (2002). Herbal remedies. New Engl J Med, 347: 2046-2056 12490687 10.1056/NEJMra020398

[27] Shibata S, Fujita M, Itokawa H, Tanaka O, Ishii T (1963). Studies on the constituents of japanese and chinese crude drugs. Xi. Panaxadiol, a sapogenin of ginseng roots. Chem Pharm Bull, 11: 759-761 14068710 10.1248/cpb.11.759

[28] Lu JM, Yao Q, Chen C (2009). Ginseng compounds: an update on their molecular mechanisms and medical applications. Curr Vasc Pharmacol, 7: 293-302 19601854 10.2174/157016109788340767 PMC2928028

[29] Shergis JL, Zhang AL, Zhou W, Xue CC (2013). Panax ginseng in randomised controlled trials: a systematic review. Phytother Res, 27: 949-965 22969004 10.1002/ptr.4832

[30] Matsuura H, Kasai R, Tanaka O, Saruwatari Y, Kunihiro K, Fuwa T (1984). Further studies on the dammarane-saponins of ginseng roots. Chem Pharm Bull (Tokyo), 32: 1188-1192

[31] Ryu JH, Park JH, Eun JH, Jung JH, Dong HS (1997). A dammarane glycoside from Korean red ginseng. Phytochemistry, 44: 931-933

[32] Namba T, Matsushige K, Morita T, Tanaka O (1986). Saponins of plants of Panax species collected in Central Nepal and their chemotaxonomical significance. Chem Pharm Bull (Tokyo), 34: 730-738 10.1248/cpb.48.889 10866157

[33] Sanada S KN, Shoji J, Tanaka O, Shibata S (1974). Studies on the saponin of ginseng. I. Structures of ginsenoside-Ro, -Rb1, -Rc, and -Rd. Chem Pharm Bull (Tokyo). 22: 421-428

[34] Lim W, Mudge KW, Vermeylen F (2005). Effects of Population, Age, and Cultivation Methods on Ginsenoside Content of Wild American Ginseng (Panax quinquefolium). J Agri Food Chem, 53: 8498-8505 10.1021/jf051070y 16248544

[35] Schlag EM, Mcintosh MS (2006). Ginsenoside content and variation among and within American ginseng (Panax quinquefolius L.) populations. Phytochemistry, 67: 1510-1519 16839573 10.1016/j.phytochem.2006.05.028

[36] Li W, Gu C, Zhang H, Awang DV, Fitzloff JF, Fong HH, et al (2000). Use of high-performance liquid chromatography-tandem mass spectrometry to distinguish Panax ginseng C. A. Meyer (Asian ginseng) and Panax quinquefolius L. (North American ginseng). Anal Chemi, 72: 5417-5422 10.1021/ac000650l 11080895

[37] Assinewe VA, Baum BR., Gagnon D, Arnason JT (2003). Phytochemistry of Wild Populations of Panax quinquefolius L. (North American Ginseng). J Agri Food Chem, 51: 4549-4553 10.1021/jf030042h 14705875

[38] Bae EA, Han MJ, Kim EJ, Kim DH (2004). Transformation of ginseng saponins to ginsenoside Rh2 by acids and human intestinal bacteria and biological activities of their transformants. Arch of Pharm Res, 27: 61-67 14969341 10.1007/BF02980048

[39] Liu Y, Zhang JW, Li W, Ma H, Sun J, Deng MC, et al (2006). Ginsenoside metabolites, rather than naturally occurring ginsenosides, lead to inhibition of human cytochrome P450 enzymes. Toxicol Sci, 91: 356-364 16547074 10.1093/toxsci/kfj164

[40] Kim MS (2013). Korean Red Ginseng Tonic Extends Lifespan in D. melanogaster. Biomol Ther (Seoul), 21: 241-245 24265871 10.4062/biomolther.2013.024 PMC3830124

[41] Lee JH, Choi SH, Kwon OS, Shin TJ, Lee JH, Lee BH, et al (2007). Effects of ginsenosides, active ingredients of Panax ginseng, on development, growth, and life span of Caenorhabditis elegans. Biol Pharm Bull, 30: 2126-2134 17978487 10.1248/bpb.30.2126

[42] Bittles AH, Fulder SJ, Grant EC, Nicholls MR (1979). The effect of ginseng on lifespan and stress responses in mice. Gerontology, 25: 125-131 571386 10.1159/000212330

[43] Miller SC, Delorme D, Shan JJ (2011). Extract of North American ginseng (Panax quinquefolius), administered to leukemic, juvenile mice extends their life span. J Complement Integr Med, 8 10.2202/1553-3840.1315 22754952

[44] Camici GG, Shi Y, Cosentino F, Francia P, Luscher TF (2011). Anti-aging medicine: molecular basis for endothelial cell-targeted strategies - a mini-review. Gerontology, 57: 101-108 20431281 10.1159/000314227

[45] Ramesh T, Kim SW, Hwang SY, Sohn SH, Yoo SK, Kim SK (2012). Panax ginseng reduces oxidative stress and restores antioxidant capacity in aged rats. Nutr Res, 32: 718-726 23084645 10.1016/j.nutres.2012.08.005

[46] Ramesh T, Kim SW, Sung JH, Hwang SY, Sohn SH, Yoo SK, et al (2012). Effect of fermented Panax ginseng extract (GINST) on oxidative stress and antioxidant activities in major organs of aged rats. Exp Gerontol, 47: 77-84 22075532 10.1016/j.exger.2011.10.007

[47] Kim HG, Yoo SR, Park HJ, Lee NH, Shin JW, Sathyanath R, et al (2011). Antioxidant effects of Panax ginseng C.A. Meyer in healthy subjects: a randomized, placebo-controlled clinical trial. Food Chem Toxicol, 49: 2229-2235 21699953 10.1016/j.fct.2011.06.020

[48] Doh KC, Lim SW, Piao SG, Jin L, Heo SB, Zheng YF, et al (2013). Ginseng treatment attenuates chronic cyclosporine nephropathy via reducing oxidative stress in an experimental mouse model. Am J Nephrol, 37: 421-433 23594788 10.1159/000349921

[49] Seo SJ, Cho JY, Jeong YH, Choi YS (2013). Effect of Korean red ginseng extract on liver damage induced by short-term and long-term ethanol treatment in rats. J Ginseng Res, 37: 194-200 23717172 10.5142/jgr.2013.37.194 PMC3659632

[50] Stangel M, Mix E, Zettl UK, Gold R (2001). Oxides and apoptosis in inflammatory myopathies. Microsc Res Tech, 55: 249-258 11748863 10.1002/jemt.1174

[51] Voces J, Cabral de Oliveira AC, Prieto JG, Vila L, Perez AC, Duarte ID, et al (2004). Ginseng administration protects skeletal muscle from oxidative stress induced by acute exercise in rats. Braz J Med Biol Res, 37: 1863-1871 15558193 10.1590/s0100-879x2004001200012

[52] Lee NJ, Lee JW, Sung JH, Ko YG, Hwang S, Kang JK (2013). Effects of administration of IH901, a ginsenoside intestinal metabolite, on muscular and pulmonary antioxidant functions after eccentric exercise. J Vet Sci, 14: 249-256 23820200 10.4142/jvs.2013.14.3.249 PMC3788149

[53] He Z, Wang X, Li G, Zhao Y, Zhang J, Niu C, et al (2015). Antioxidant activity of prebiotic ginseng polysaccharides combined with potential probiotic Lactobacillus plantarum C88. Int J Food Sci Tech, 50: 1673-1682

[54] Lee LS, Cho CW, Hong HD, Lee YC, Choi UK, Kim YC (2013). Hypolipidemic and antioxidant properties of phenolic compound-rich extracts from white ginseng (Panax ginseng) in cholesterol-fed rabbits. Molecules, 18: 12548-12560 24152674 10.3390/molecules181012548 PMC6269857

[55] Jiang R, Sun L, Wang Y, Liu J, Liu X, Feng H, et al (2014). Chemical composition, and cytotoxic, antioxidant and antibacterial activities of the essential oil from ginseng leaves. Nat Prod Commun, 9: 865-868 25115102

[56] Dong GZ, Jang EJ, Kang SH, Cho IJ, Park SD, Kim SC, et al (2013). Red ginseng abrogates oxidative stress via mitochondria protection mediated by LKB1-AMPK pathway. BMC Complement Altern Med, 13: 64 23506615 10.1186/1472-6882-13-64 PMC3635924

[57] Zhuang CL, Mao XY, Liu S, Chen WZ, Huang DD, Zhang CJ, et al (2014). Ginsenoside Rb1 improves postoperative fatigue syndrome by reducing skeletal muscle oxidative stress through activation of the PI3K/Akt/Nrf2 pathway in aged rats. Eur J Pharmacol, 740: 480-487 24975098 10.1016/j.ejphar.2014.06.040

[58] Ferrari AU, Radaelli A, Centola M (2003). Invited Review: Aging and the cardiovascular system. J Appl Physiol, 95: 2591-2597 14600164 10.1152/japplphysiol.00601.2003

[59] Pearson AC, Gudipati CV, Labovitz AJ (1991). Effects of aging on left ventricular structure and function. Am Heart J, 121: 871-875 1825740 10.1016/0002-8703(91)90201-r

[60] Yuan SM (2015). Potential cardioprotective effects of Ginseng preparations. Pak J Pharm Sci, 28: 963-968 26004730

[61] Xu K, Zhang Y, Wang Y, Ling P, Xie X, Jiang C, et al (2014). Ginseng Rb fraction protects glia, neurons and cognitive function in a rat model of neurodegeneration. PLoS One, 9: e101077 24971630 10.1371/journal.pone.0101077 PMC4074135

[62] Lin JW, Cherng YG, Chen LJ, Niu HS, Chang CK, Niu CS (2014). Ginseng is useful to enhance cardiac contractility in animals. Biomed Res Int, 2014: 723084 10.1155/2014/723084 PMC3932289 24689053

[63] Wu Y, Lu X, Xiang FL, Lui EM, Feng Q (2011). North American ginseng protects the heart from ischemia and reperfusion injury via upregulation of endothelial nitric oxide synthase. Pharmacol Res, 64: 195-202 21621617 10.1016/j.phrs.2011.05.006

[64] Jovanovski E, Jenkins A, Dias AG, Peeva V, Sievenpiper J, Arnason JT, et al (2010). Effects of Korean red ginseng (Panax ginseng C.A. Mayer) and its isolated ginsenosides and polysaccharides on arterial stiffness in healthy individuals. Am J Hypertens, 23: 469-472 20134405 10.1038/ajh.2010.5

[65] Knight J, Nigam Y (2008). Exploring the anatomy and physiology of ageing. Part 1--The cardiovascular system. Nurs Times, 104: 26-27 18727348

[66] Jovanovski E, Peeva V, Sievenpiper JL, Jenkins AL, Desouza L, Rahelic D, et al (2014). Modulation of endothelial function by Korean red ginseng (Panax ginseng C.A. Meyer) and its components in healthy individuals: a randomized controlled trial. Cardiovasc Ther, 32: 163-169 24758417 10.1111/1755-5922.12077

[67] Choi K, Yoon J, Lim HK, Ryoo S (2014). Korean red ginseng water extract restores impaired endothelial function by inhibiting arginase activity in aged mice. Korean J Physiol Pharmacol, 18: 95-101 24757370 10.4196/kjpp.2014.18.2.95 PMC3994309

[68] Wongrakpanich S, Petchlorlian A, Rosenzweig A (2016). Sensorineural Organs Dysfunction and Cognitive Decline: A Review Article. Aging Dis, 7: 763-769 28053826 10.14336/AD.2016.0515 PMC5198866

[69] Mattson MP, Magnus T (2006). Ageing and neuronal vulnerability. Nat Rev Neurosci, 7: 278-294 16552414 10.1038/nrn1886 PMC3710114

[70] Ong WY, Farooqui T, Koh HL, Farooqui AA, Ling EA (2015). Protective effects of ginseng on neurological disorders. Front Aging Neurosci, 7: 129 26236231 10.3389/fnagi.2015.00129 PMC4503934

[71] Li SC, Lindenberger U, Sikstrom S (2001). Aging cognition: from neuromodulation to representation. Trends Cogn Sci, 5: 479-486 11684480 10.1016/s1364-6613(00)01769-1

[72] Rapp PR, Gallagher M (1996). Preserved neuron number in the hippocampus of aged rats with spatial learning deficits. Proc Natl Acad Sci U S A, 93: 9926-9930 8790433 10.1073/pnas.93.18.9926 PMC38531

[73] Zhong YM, Nishijo H, Uwano T, Tamura R, Kawanishi K, Ono T (2000). Red ginseng ameliorated place navigation deficits in young rats with hippocampal lesions and aged rats. Physiol Behav, 69: 511-525 10913791 10.1016/s0031-9384(00)00206-7

[74] Lee Y, Oh S (2015). Administration of red ginseng ameliorates memory decline in aged mice. J Ginseng Res, 39: 250-256 26199557 10.1016/j.jgr.2015.01.003 PMC4506366

[75] D'Angelo L, Grimaldi R, Caravaggi M, Marcoli M, Perucca E, Lecchini S, et al (1986). A double-blind, placebo-controlled clinical study on the effect of a standardized ginseng extract on psychomotor performance in healthy volunteers. J Ethnopharmacol, 16: 15-22 3528672 10.1016/0378-8741(86)90063-2

[76] Petkov VD, Mosharrof AH (2010). Age- and individual-related specificities in the effects of standardized ginseng extract on learning and memory (experiments on rats). Phytother Res, 1: 80-84

[77] Cheng Y, Shen LH, Zhang JT (2005). Anti-amnestic and anti-aging effects of ginsenoside Rg1 and Rb1 and its mechanism of action. Acta Pharmacol Sin, 26: 143-149 15663889 10.1111/j.1745-7254.2005.00034.x

[78] Zhang JT, Liu Y, Qu ZW, Zhang XL, Xiao HL (1988). Influence of ginsenoside Rb1 and Rg1 on some central neurotransmitter receptors and protein biosynthesis in the mouse brain. Yao Xue Xue Bao, 23: 12-16 2840797

[79] Zhang JT, Qu ZW, Liu Y, Deng HL (1990). Preliminary study on antiamnestic mechanism of ginsenoside Rg1 and Rb1. Chin Med J (Engl), 103: 932-938 2177392

[80] Lu JM, Yao Q, Chen C (2009). Ginseng Compounds: An Update on Their Molecular Mechanisms and Medical Applications. Curr Vasc Pharmacol, 7(3): 293-302 19601854 10.2174/157016109788340767 PMC2928028

[81] Al-Hazmi MA, Rawi SM, Arafa NM, Wagas A, Montasser AO (2015). The potent effects of ginseng root extract and memantine on cognitive dysfunction in male albino rats. Toxicol Ind Health, 31: 494-509 23406953 10.1177/0748233713475517

[82] Shin TJ, Kim HJ, Kwon BJ, Choi SH, Kim HB, Hwang SH, et al (2012). Gintonin, a ginseng-derived novel ingredient, evokes long-term potentiation through N-methyl-D-aspartic acid receptor activation: involvement of LPA receptors. Mol Cells, 34: 563-572 23161173 10.1007/s10059-012-0254-4 PMC3887827

[83] Shen LH, Zhang JT (2004). Ginsenoside Rg1 promotes proliferation of hippocampal progenitor cells. Neurol Res, 26: 422-428 15198871 10.1179/016164104225016047

[84] Zhu J, Mu X, Zeng J, Xu C, Liu J, Zhang M, et al (2014). Ginsenoside Rg1 prevents cognitive impairment and hippocampus senescence in a rat model of D-galactose-induced aging. PLoS One, 9: e101291 24979747 10.1371/journal.pone.0101291 PMC4076296

[85] Yang L, Zhang J, Zheng K, Shen H, Chen X (2014). Long-term ginsenoside Rg1 supplementation improves age-related cognitive decline by promoting synaptic plasticity associated protein expression in C57BL/6J mice. J Gerontol A Biol Sci Med Sci, 69: 282-294 23833204 10.1093/gerona/glt091

[86] Shi YQ, Huang TW, Chen LM, Pan XD, Zhang J, Zhu YG, et al (2010). Ginsenoside Rg1 attenuates amyloid-beta content, regulates PKA/CREB activity, and improves cognitive performance in SAMP8 mice. J Alzheimers Dis, 19: 977-989 20157253 10.3233/JAD-2010-1296

[87] Ni N, Liu Q, Ren H, Wu D, Luo C, Li P, et al (2014). Ginsenoside Rb1 protects rat neural progenitor cells against oxidative injury. Molecules, 19: 3012-3024 24662068 10.3390/molecules19033012 PMC6271345

[88] Liao B, Newmark H, Zhou R (2002). Neuroprotective effects of ginseng total saponin and ginsenosides Rb1 and Rg1 on spinal cord neurons in vitro. Exp Neurol, 173: 224-234 11822886 10.1006/exnr.2001.7841

[89] Wang B, Feng G, Tang C, Wang L, Cheng H, Zhang Y, et al (2013). Ginsenoside Rd maintains adult neural stem cell proliferation during lead-impaired neurogenesis. Neurol Sci, 34: 1181-1188 23073826 10.1007/s10072-012-1215-6

[90] Qian YH, Han H, Hu XD, Shi LL (2009). Protective effect of ginsenoside Rb1 on beta-amyloid protein(1-42)-induced neurotoxicity in cortical neurons. Neurol Res, 31: 663-667 19138476 10.1179/174313209X385572

[91] Bak DH, Kim HD, Kim YO, Park CG, Han SY, Kim JJ (2016). Neuroprotective effects of 20(S)-protopanaxadiol against glutamate-induced mitochondrial dysfunction in PC12 cells. Int J Mol Med, 37: 378-386 26709399 10.3892/ijmm.2015.2440 PMC4716797

[92] Kim EH, Kim IH, Lee MJ, Thach Nguyen C, Ha JA, Lee SC, et al (2013). Anti-oxidative stress effect of red ginseng in the brain is mediated by peptidyl arginine deiminase type IV (PADI4) repression via estrogen receptor (ER) beta up-regulation. J Ethnopharmacol, 148: 474-485 23665163 10.1016/j.jep.2013.04.041

[93] Li H, Kang T, Qi B, Kong L, Jiao Y, Cao Y, et al (2016). Neuroprotective effects of ginseng protein on PI3K/Akt signaling pathway in the hippocampus of D-galactose/AlCl3 inducing rats model of Alzheimer's disease. J Ethnopharmacol, 179: 162-169 26721223 10.1016/j.jep.2015.12.020

[94] Shi S, Shi R, Hashizume K (2012). American ginseng improves neurocognitive function in senescence-accelerated mice: possible role of the upregulated insulin and choline acetyltransferase gene expression. Geriatr Gerontol Int, 12: 123-130 21702872 10.1111/j.1447-0594.2011.00719.x

[95] Rinwa P, Kumar A (2014). Modulation of nitrergic signalling pathway by American ginseng attenuates chronic unpredictable stress-induced cognitive impairment, neuroinflammation, and biochemical alterations. Naunyn Schmiedebergs Arch Pharmacol, 387: 129-141 24132508 10.1007/s00210-013-0925-5

[96] Shin K, Guo H, Cha Y, Ban YH, Seo da W, Choi Y, et al (2016). Cereboost, an American ginseng extract, improves cognitive function via up-regulation of choline acetyltransferase expression and neuroprotection. Regul Toxicol Pharmacol, 78: 53-58 27112419 10.1016/j.yrtph.2016.04.006

[97] Liu D, Li B, Liu Y, Attele AS, Kyle JW, Yuan CS (2001). Voltage-dependent inhibition of brain Na(+) channels by American ginseng. Eur J Pharmacol, 413: 47-54 11173062 10.1016/s0014-2999(01)00735-x

[98] Itoh T, Zang YF, Murai S, Saito H (1989). Effects of Panax ginseng root on the vertical and horizontal motor activities and on brain monoamine-related substances in mice. Planta Med, 55: 429-433 2813579 10.1055/s-2006-962058

[99] Watanabe H, Ohta H, Imamura L, Asakura W, Matoba Y, Matsumoto K (1991). Effect of Panax ginseng on age-related changes in the spontaneous motor activity and dopaminergic nervous system in the rat. Jpn J Pharmacol, 55: 51-56 1674972 10.1254/jjp.55.51

[100] Cabral de Oliveira AC, Perez AC, Merino G, Prieto JG, Alvarez AI (2001). Protective effects of Panax ginseng on muscle injury and inflammation after eccentric exercise. Comp Biochem Physiol C Toxicol Pharmacol, 130: 369-377 11701393 10.1016/s1532-0456(01)00262-9

[101] Jung HL, Kwak HE, Kim SS, Kim YC, Lee CD, Byurn HK, et al (2011). Effects of Panax ginseng supplementation on muscle damage and inflammation after uphill treadmill running in humans. Am J Chin Med, 39: 441-450 21598413 10.1142/S0192415X11008944

[102] Callaghan TM, Wilhelm KP (2008). A review of ageing and an examination of clinical methods in the assessment of ageing skin. Part 2: Clinical perspectives and clinical methods in the evaluation of ageing skin. Int J Cosmet Sci, 30: 323-332 18822037 10.1111/j.1468-2494.2008.00455.x

[103] Hwang E, Park SY, Jo H, Lee DG, Kim HT, Kim YM, et al (2015). Efficacy and Safety of Enzyme-Modified Panax ginseng for Anti-Wrinkle Therapy in Healthy Skin: A Single-Center, Randomized, Double-Blind, Placebo-Controlled Study. Rejuvenation Res, 18: 449-457 25867599 10.1089/rej.2015.1660

[104] Cho S, Won CH, Lee DH, Lee MJ, Lee S, So SH, et al (2009). Red ginseng root extract mixed with Torilus fructus and Corni fructus improves facial wrinkles and increases type I procollagen synthesis in human skin: a randomized, double-blind, placebo-controlled study. J Med Food, 12: 1252-1259 20041778 10.1089/jmf.2008.1390

[105] Lee HS, Kim MR, Park Y, Park HJ, Chang UJ, Kim SY, et al (2012). Fermenting red ginseng enhances its safety and efficacy as a novel skin care anti-aging ingredient: in vitro and animal study. J Med Food, 15: 1015-1023 23126662 10.1089/jmf.2012.2187 PMC3491619

[106] Lee HJ, Kim JS, Song MS, Seo HS, Moon C, Kim JC, et al (2009). Photoprotective effect of red ginseng against ultraviolet radiation-induced chronic skin damage in the hairless mouse. Phytother Res, 23: 399-403 18979525 10.1002/ptr.2640

[107] Kim YG, Sumiyoshi M, Sakanaka M, Kimura Y (2009). Effects of ginseng saponins isolated from red ginseng on ultraviolet B-induced skin aging in hairless mice. Eur J Pharmacol, 602: 148-156 19041641 10.1016/j.ejphar.2008.11.021

[108] Kang TH, Park HM, Kim YB, Kim H, Kim N, Do JH, et al (2009). Effects of red ginseng extract on UVB irradiation-induced skin aging in hairless mice. J Ethnopharmacol, 123: 446-451 19501277 10.1016/j.jep.2009.03.022

[109] Oh SJ, Lee S, Choi WY, Lim CJ (2014). Skin anti-photoaging properties of ginsenoside Rh2 epimers in UV-B-irradiated human keratinocyte cells. J Biosci, 39: 673-682 25116621 10.1007/s12038-014-9460-x

[110] Kim S, Kang BY, Cho SY, Sung DS, Shin ES, Chang HK, et al(2004) 20-O-β-D-Glucopyranosyl-20 (S)-protopanaxadiol (compound K) induces expression of hyaluronan synthase 2 gene in transformed human keratinocytes and fibroblasts and increases hyaluronan in hairless mouse skin. Int J Cosmet Sci, 26: 317-318 10.1016/j.bbrc.2004.02.046 15020224

[111] Lim TG, Jeon AJ, Yoon JH, Song D, Kim JE, Kwon JY, et al (2015). 20-O-beta-D-glucopyranosyl-20(S)-protopanaxadiol, a metabolite of ginsenoside Rb1, enhances the production of hyaluronic acid through the activation of ERK and Akt mediated by Src tyrosin kinase in human keratinocytes. Int J Mol Med, 35: 1388-1394 25738334 10.3892/ijmm.2015.2121

[112] Kim MR, Lee HS, Choi HS, Kim SY, Park Y, Suh HJ (2014). Protective effects of ginseng leaf extract using enzymatic extraction against oxidative damage of UVA-irradiated human keratinocytes. Appl Biochem Biotechnol, 173: 933-945 24736942 10.1007/s12010-014-0886-2

[113] Kim J, Cho SY, Kim SH, Cho D, Kim S, Park CW, et al (2016). Effects of Korean ginseng berry on skin antipigmentation and antiaging via FoxO3a activation. J Ginseng Res: 1-7 28701867 10.1016/j.jgr.2016.05.005 PMC5489743

[114] Nigam Y, Knight J (2008). Exploring the anatomy and physiology of ageing. Part 9--The immune system. Nurs Times, 104: 58-59 19068889

[115] Cho YJ, Son HJ, Kim KS (2014). A 14-week randomized, placebo-controlled, double-blind clinical trial to evaluate the efficacy and safety of ginseng polysaccharide (Y-75). J Transl Med, 12: 283 25297058 10.1186/s12967-014-0283-1 PMC4196019

[116] Azike CG, Charpentier PA, Lui EM (2015). Stimulation and suppression of innate immune function by American ginseng polysaccharides: biological relevance and identification of bioactives. Pharm Res, 32: 876-897 25208875 10.1007/s11095-014-1503-3

[117] Liu J, Wang S, Liu H, Yang L, Nan G (1995). Stimulatory effect of saponin from Panax ginseng on immune function of lymphocytes in the elderly. Mech Ageing Dev, 83: 43-53 8523901 10.1016/0047-6374(95)01618-a

[118] Shin HY, Jeong HJ, Hong SH, Um JY, Shin TY, Kwon SJ, et al (2006). The effect of Panax ginseng on forced immobility time & immune function in mice. Indian J Med Res, 124: 199-206 17015935

[119] Yu X, Yang X, Cui B, Wang L, Ren G (2014). Antioxidant and immunoregulatory activity of alkali-extractable polysaccharides from North American ginseng. Int J Biol Macromol, 65: 357-361 24472508 10.1016/j.ijbiomac.2014.01.046

[120] Lemmon HR, Sham J, Chau LA, Madrenas J (2012). High molecular weight polysaccharides are key immunomodulators in North American ginseng extracts: characterization of the ginseng genetic signature in primary human immune cells. J Ethnopharmacol, 142: 1-13 22521964 10.1016/j.jep.2012.04.004

[121] Wang Y, Liu Y, Zhang XY, Xu LH, Ouyang DY, Liu KP, et al (2014). Ginsenoside Rg1 regulates innate immune responses in macrophages through differentially modulating the NF-kappaB and PI3K/Akt/mTOR pathways. Int Immunopharmacol, 23: 77-84 25179784 10.1016/j.intimp.2014.07.028

[122] Kim J, Ahn H, Han BC, Lee SH, Cho YW, Kim CH, et al (2014). Korean red ginseng extracts inhibit NLRP3 and AIM2 inflammasome activation. Immunol Lett, 158: 143-150 24418475 10.1016/j.imlet.2013.12.017

[123] Yu ZP, Xu DD, Lu LF, Zheng XD, Chen W (2016). Immunomodulatory effect of a formula developed from American ginseng and Chinese jujube extracts in mice. J Zhejiang Univ Sci B, 17: 147-157 26834015 10.1631/jzus.B1500170 PMC4757584

[124] Jia ZY, Xie X, Wang XY, Jia W (2014). Comparative study of main components of ginseng on immune function of rats. Zhongguo Zhong Yao Za Zhi, 39:3363-3366 25522629

[125] Tran TL, Kim YR, Yang JL, Oh DR, Dao TT, Oh WK (2014). Dammarane triterpenes from the leaves of Panax ginseng enhance cellular immunity. Bioorg Med Chem, 22: 499-504 24290061 10.1016/j.bmc.2013.11.002

[126] Byeon SE, Lee J, Kim JH, Yang WS, Kwak YS, Kim SY, et al (2012). Molecular mechanism of macrophage activation by red ginseng acidic polysaccharide from Korean red ginseng. Mediators Inflamm, 2012: 732860 10.1155/2012/732860 PMC3306998 22474399

[127] Chung HY, Cesari M, Anton S, Marzetti E, Giovannini S, Seo AY, et al (2009). Molecular inflammation: underpinnings of aging and age-related diseases. Ageing Res Rev, 8: 18-30 18692159 10.1016/j.arr.2008.07.002 PMC3782993

[128] Hong CE, Lyu SY (2011). Anti-inflammatory and Anti-oxidative Effects of Korean Red Ginseng Extract in Human Keratinocytes. Immune Netw, 11: 42-49 21494373 10.4110/in.2011.11.1.42 PMC3072674

[129] Jin Y, Kotakadi VS, Ying L, Hofseth AB, Cui X, Wood PA, et al (2008). American ginseng suppresses inflammation and DNA damage associated with mouse colitis. Carcinogenesis, 29: 2351-2359 18802031 10.1093/carcin/bgn211 PMC2639244

[130] Jin Y, Hofseth AB, Cui X, Windust AJ, Poudyal D, Chumanevich AA, et al (2010). American ginseng suppresses colitis through p53-mediated apoptosis of inflammatory cells. Cancer Prev Res (Phila), 3: 339-347 20179294 10.1158/1940-6207.CAPR-09-0116 PMC2833220

[131] Qu C, Li B, Lai Y, Li H, Windust A, Hofseth LJ, et al (2015). Identifying panaxynol, a natural activator of nuclear factor erythroid-2 related factor 2 (Nrf2) from American ginseng as a suppressor of inflamed macrophage-induced cardiomyocyte hypertrophy. J Ethnopharmacol, 168: 326-336 25882312 10.1016/j.jep.2015.04.004 PMC4810680

[132] Lee J, Cho JY, Kim WK (2014). Anti-inflammation effect of Exercise and Korean red ginseng in aging model rats with diet-induced atherosclerosis. Nutr Res Pract, 8: 284-291 24944773 10.4162/nrp.2014.8.3.284 PMC4058562

[133] Kim CK, Cho DH, Lee KS, Lee DK, Park CW, Kim WG, et al (2012). Ginseng Berry Extract Prevents Atherogenesis via Anti-Inflammatory Action by Upregulating Phase II Gene Expression. Evid Based Complement Alternat Med, 2012: 490301 10.1155/2012/490301 PMC3519292 23243449

